# Dentistry Practice—Kind of Certificates Required in Maryland

**Published:** 1898-06

**Authors:** 


### Dentistry Practice—Kind of Certificates Required in Maryland.

An interesting point of statutory construction is involved in the decision of the Court of Appeals of Maryland, which was handed down February 10, 1898, in the case of Knowles vs. State. An 1896 act of that State declares that it shall be unlawful for any person to practice dentistry in the State without first obtaining a certificate as provided by the act, while section 12 thereof excepts from its operation certain classes of persons, providing, among other things, that nothing therein shall be construed as to interfere "with persons holding certificates issued to them prior to the passage of this act." In this case, after a conviction in the criminal court of Baltimore City for unlawfully practicing dentistry without having obtained from the State Board of Dental Examiners a certificate as required by the foregoing act, appeal was taken, contention being urged that the trial court had erred in rejecting as evidence a certificate of qualification and registration issued by the Board of Dental Examiners of the State of Ohio. But the Court of Appeals of Maryland takes the position that it was the intention of the Legislature to confine the act of 1896 in its application to certificates which had been issued by the Dental Board of that State, under article 32 of the Code of Public General Laws, and to save all certificates which had been previously issued thereunder, without giving the act any extra-territorial force, especially as its language, taken in connection with the previous legislation on the subject, shows conclusively that the act refers only to certificates issued by the Board of Dental Examiners of Maryland, and not to those issued by other States. For these reasons, it holds that there was no error in the rejection of the testimony offered in presenting the Ohio certificate.

